# Current Status and Future Direction of the High‐Temperature Resistance in Wheat to Control Stripe Rust

**DOI:** 10.1111/mpp.70192

**Published:** 2025-12-17

**Authors:** Xinqian Lyu, Guocheng Li, Chang Su, Xujun Luo, Yuankang Zhu, Tao Liu, Yuxiang Li, Xiaoping Hu, Chengyun Li, Yangshan Hu

**Affiliations:** ^1^ State Key Laboratory for Conservation and Utilization of Bio‐Resources in Yunnan and College of Plant Protection Yunnan Agricultural University Kunming Yunnan China; ^2^ Ministry of Agriculture and Rural Affairs (MARA) Key Laboratory of Sustainable Crop Production in the Middle Reaches of the Yangtze River (Co‐Construction by Ministry and Province), College of Agriculture Yangtze University Jingzhou China; ^3^ School of Agriculture Ningxia University Yinchuan Ningxia China; ^4^ Key Laboratory of Plant Protection Resources and Pest Management, Ministry of Education; Key Laboratory of Integrated Pest Management on Crops in Northwestern Loess Plateau, Ministry of Agriculture and Rural Affairs; College of Plant Protection Northwest A&F University Yangling Shaanxi China

**Keywords:** high‐temperature resistance, molecular mechanism, *Puccinia striiformis* f. sp. *tritici*, quantitative trait loci, wheat ( *Triticum aestivum* L.) stripe rust, *Yr* genes

## Abstract

This review summarises the current advances and future perspectives on high‐temperature resistance in wheat against stripe rust, caused by the airborne fungal pathogen *Puccinia striiformis* f. sp. *tritici* (Pst). High‐temperature resistance, comprising high‐temperature adult‐plant (HTAP) and high‐temperature all‐stage (HTAS) resistance, is critical for durable disease control. HTAP resistance occurs in adult plants at relatively high temperatures, whereas HTAS resistance acts across all growth stages at relatively high temperatures. Genetic mapping has identified numerous HTAP‐resistance genes and quantitative trait loci (QTLs) in various chromosomal regions, especially in the B subgenome. Cloned genes, such as *Yr18*, *Yr36* and *Yr46*, reveal mechanisms involving cell necrosis induction, photosynthesis modulation and nutrient transportation. For HTAS resistance, defence‐related factors such as nucleotide‐binding site‐leucine‐rich repeat proteins, transcription factors and receptor‐like kinases that mediate resistance via plant–pathogen interactions have been identified; however, research on identifying the genes or QTLs that can be used in breeding programmes is limited. To overcome host defence, Pst secretes effectors that suppress high‐temperature resistance by targeting host proteins. Future research should focus on novel gene mining, regulatory network deciphering, effector–host interaction studies and breeding applications via marker‐assisted selection and gene editing.

## Graphical abstract

High‐temperature resistance: A gentle might to cradle wheat against stripe rust—it draws heat's strength, spanning growth stages, outpacing pathogen shifts, a shield for fields in a warming world.

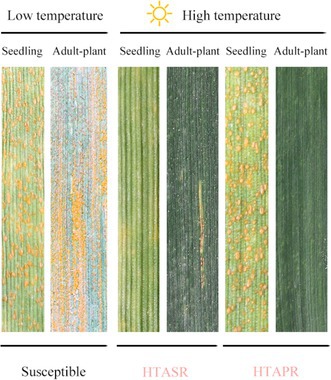

## 1 Introduction

Wheat (*Triticum* spp.), especially common wheat ( *T. aestivum* ), is one of the most widely cultivated staple food crops and the leading source of plant protein for humans. In response to the continuous decrease in wheat‐cultivated areas and the growth in population size, the grain yield of wheat must grow at an average annual rate of 2% (Gill et al. 2004). However, wheat production is severely threatened by biotic stresses, including rusts, powdery mildew and Fusarium head blight. Stripe (yellow) rust, caused by the airborne obligate biotrophic fungus *Puccinia striiformis* f. sp. *tritici* (Pst), can substantially reduce the grain yield and quality of wheat. Stripe rust occurs in most of the major wheat‐producing countries, including China, the United States, India, Australia, Pakistan and European countries (Line 2002). The yield loss caused by stripe rust is 0.5%–5% in ordinary years, and in epidemic years, it can reach 5%–25% in a country or even result in total crop failure in a field (Wan et al. 2004; Wellings 2011). The annual economic losses caused by stripe rust almost reach $10 billion (Beddow et al. 2015).

Rust fungi typically have complex life cycles. There are five spore forms for wheat stripe rust pathogen Pst, that is, pycniospore, aecidiospore, urediniospore, teliospore and basidiospore (Hovmoller et al. 2011). Pst is a heteroecious, macrocyclic fungus. *Berberis* spp. and *Mahonia aquifolium* serve as alternative hosts for completing the life cycle of Pst (Jin et al. 2010; Li, Chen, et al. 2021; Wang and Chen 2013; Zhao et al. 2016). However, most infections of Pst on alternate hosts occur under artificial conditions, whereas natural infection of *Berberis* spp. has only been reported in China (Li, Chen, et al. 2021). Owing to mutations, somatic recombination and sexual reproduction, the frequent virulence variation in Pst has led to the overcoming of resistance in existing resistant cultivars. Identifying genes with broad‐spectrum and stable resistance has become an urgent issue in global wheat production. In recent years, global climate change has led to frequent high temperatures during the late growth stages of wheat. Traditional race‐specific resistance struggles to meet the demand for durable disease control, as the widespread deployment of a single *R* gene imposes selection pressure that enables Pst races to overcome the *R*‐gene‐mediated defence (Schwessinger 2017). In contrast, high‐temperature resistance exhibits both temperature dependence and race non‐specificity, and has thus emerged as a core strategy for the sustainable management of wheat stripe rust (Chen 2020; Zhu et al. 2021). Therefore, this review focuses on durable and stable high‐temperature resistance to Pst in wheat and discusses research progress and application prospects.

## 2 Current Status of High‐Temperature Resistance to Pst

### 2.1 Overview of Wheat Resistance to Pst

Based on the growth stage of wheat, resistance to Pst can be divided into all‐stage resistance (ASR) and adult‐plant resistance (APR) (Chen 2005). As the name suggests, ASR refers to resistance throughout the growth stages; however, it is usually race‐specific and controlled by a single gene. To date, over 70% of the 87 permanently named *Yr* genes confer ASR (Table 1), and most have become ineffective against the current predominant races of Pst (Hulbert and Pumphrey 2014; Klymiuk et al. 2022; Sharma et al. 2024). For example, race CYR29, which has virulence to *Yr9*, contributed to the epidemic of Pst in 1990 in China (Chen 2005; Wan et al. 2007); CYR34 can infect wheat cultivars carrying *Yr24/26*, which have been widely planted in recent years in China (Liu et al. 2017); breakdown of *Yr17* was first detected in the United Kingdom and Denmark in 1994, spread rapidly to other European countries within 4 years, with virulent race frequencies exceeding 70% (Bayles et al. 2000), and prevalent race CYR31 and CYR32 in China has also overcome *Yr17* (Gebreslasie et al. 2020); the epidemics of stripe rust worldwide in the past decades were caused by Pst races overcoming race‐specific resistance genes such as *Yr2*, *Yr9* and *Yr27* (Chen 2020). Therefore, there is an urgent need to identify novel, broad‐spectrum and durable resistance genes.

**TABLE 1:** Characterised permanently named *Yr* genes conferring resistance to *Puccinia striiformis* f. sp. *tritici* in wheat.

Gene	Species origin	Chromosome	Cultivar/Line	Flanking markers	Type	Encoded protein	References
*Yr1*	*Triticum aestivum*	2AL	Chinese 166	*Xstm673acag‐Sr48*	ASR	/	Lupton and Macer (1962) Bansal et al. (2009)
*Yr2*	*T. aestivum*	7B	Heines VII	*Xwmc364*	ASR	/	Lupton and Macer (1962)
*Yr3*	*T. aestivum*	5BL	Cappelle Desprez/Hybrid 46/Minister	*Xwmc356*	ASR	/	Lupton and Macer (1962)
*Yr4*	*T. aestivum*	3BS	Cappelle Desprez/Hybrid 46	*Xcfb3530*	ASR	/	Lupton and Macer (1962)
*Yr5*	*Triticum spelta album*	2BL	*T. spelta* Album	*STS7/8‐Xbarc349*	ASR	BED‐NB‐LRR	Macer (1966) Marchal et al. (2018)
*Yr6*	*T. aestivum*	7BS	Heines Kolben	*Xgwm577*	ASR	NLR	Macer (1966) Wu et al. (2025)
*Yr7*	*Triticum durum*	2BL	Lee	*Xgwm526, Xwmc175*	ASR	BED‐NB‐LRR	Macer (1966) Marchal et al. (2018)
*Yr8*	*Aegilops comosa*	2 M/2D	Compair	*OP‐D111265*	ASR	/	Riley et al. (1968)
*Yr9*	*Secale cereale*	1RS	Clement	*AF1/AF4*	ASR	/	Macer (1975)
*Yr10*	*T. aestivum*	1BS	Moro	*Xpsp3000*	ASR	CC‐NB‐LRR	Macer (1975) Liu et al. (2014)
*Yr11*	*T. aestivum*	/	Joss Cambier	/	APR	/	McIntosh (2024)
*Yr12*	*T. aestivum*	/	Mega	/	APR	/	McIntosh (2024)
*Yr13*	*T. aestivum*	/	Maris Huntsman	/	APR	/	McIntosh (2024)
*Yr14*	*T. aestivum*	/	Hobbit	/	APR	/	McIntosh (2024)
*Yr15*	*Triticum dicoccoides*	1BS	G‐25	*Xgwm413‐Xbarc8*	APR	Kinase‐pseudokinase WTK1	Gerechter‐Amitai et al. (1989) Klymiuk et al. (2018)
*Yr16*	*T. aestivum*	2DL	Cappelle Desprez	*Xpsr641‐Xpsr681*	APR	/	Worland et al. (1986)
*Yr17*	*Aegilops ventricosa*	2AS	VPM1	*XSC‐Y15‐Xpsr150*	ASR	/	Bariana and McIntosh (1993)
*Yr18*	*T. aestivum*	7DS	Jupateco 73R	*Xgwm120‐Xgwm295*	HTAPR	ATP‐binding cassette transporter	Singh (1992) Krattinger et al. (2009)
*Yr19*	*Aegilops speltoides*	5B	Compair	/	ASR	/	Chen et al. (1995)
*Yr20*	*T. aestivum*	6D	Fielder	/	ASR	/	Chen et al. (1995)
*Yr21*	*T. aestivum*	1BL	Lemhi	*Rpslem*	ASR	/	Chen et al. (1995)
*Yr22*	*T. aestivum*	4D	Lee	/	ASR	/	Chen et al. (1995)
*Yr23*	*T. aestivum*	6D	Lee	/	ASR	/	Chen et al. (1995)
*Yr24*	*T. durum*	1BS	K773/Chuanmai 42	*Xwe173; Xgwm498‐Xbarc187*	ASR	/	McIntosh and Lagudah (2000) Li, Li, et al. (2006)
*Yr25*	*T. aestivum*	1D	TP1295	/	ASR	/	Calonnec and Johnson (1998)
*Yr26*	*Triticum turgidum*	1BS	R55	*Xgwm11/Xgwm18‐Xgwm413*	ASR	/	Ma et al. (2001)
*Yr27*	*T. aestivum*	2BS	Ciano 79	*Xcdo405‐Xbcd152*	ASR	CC‐NB‐LRR	McDonald et al. (2004) Athiyannan et al. (2022)
*Yr28*	*Aegilops tauschii*	4DS	Opata 85	*Xmwg634*	ASR	NB‐LRR	Singh et al. (2000) Zhang et al. (2019)
*Yr29*	*T. aestivum*	1BL	Pavon 76	*Xwmc44‐Xgwm140*	HTAPR	/	William et al. (2003) Rosewarne et al. (2006)
*Yr30*	*T. aestivum*	3BS	Opata 85	*AX‐108727801‐AX‐109480678*	APR	/	McIntosh (2024) Wang, Xiang, et al. (2024)
*Yr31*	*T. aestivum*	2BS	Pastor	*Lr13‐Lr23*	ASR	/	Singh et al. (2003)
*Yr32*	*T. aestivum*	2AS	Carstens V	*Xwmc198*	ASR	/	Eriksen et al. (2004)
*Yr33*	*T. aestivum*	7DL	Batavia	*Xgwm111‐Xwmc437*	ASR	/	Zahravi et al. (2003)
*Yr34*	*T. aestivum*	5AL	WAWHT2046	*Xgwm410‐B1*	HTAPR	/	Bariana et al. (2006) Chen (2013)
*Yr35*	*T. dicoccoides*	6BS	98 M71	*Xgwm191‐Lr53*	ASR	/	Marais, Pretorius, et al. (2005) Dadkhodaie et al. (2011)
*Yr36*	*T. dicoccoides*	6BS	RSL65	*Xucw71‐Xucw77/Xbarc136*	HTAPR	Kinase‐START	Uauy et al. (2005) Fu et al. (2009)
*Yr37*	*Aegilops kotschyi*	2DL	Line S14	/	ASR	/	Marais, McCallum, et al. (2005)
*Yr38*	*Aeg. sharonensis*	6AS	Line 0352–4	*Xgwm334‐Xwmc59*	ASR	/	Marais et al. (2006)
*Yr39*	*T. aestivum*	7BL	Alpowa	*Xwgp45‐Xwgp36*	HTAPR	/	Lin and Chen (2007)
*Yr40*	*Aegilops geniculata*	5DS	T5DL.5DS‐5M^g^S	*Gsp*	ASR	/	Kuraparthy et al. (2007)
*Yr41*	*T. aestivum*	2BS	Chuannong 19	*Xgwm410‐Xgwm374*	ASR	/	Luo et al. (2008)
*Yr42*	*Aegilops neglecta*	6AL	03 M119‐71A	/	ASR	/	Marais et al. (2009)
*Yr43*	*T. aestivum*	2BL	IDO377s	*Xwgp110‐Xwgp103*	ASR	/	Cheng and Chen (2010)
*Yr44*	*T. aestivum*	2BL	Zak	*XpWB5/N1R1‐Xwgp100*	ASR	/	Sui et al. (2009)
*Yr45*	*T. aestivum*	3DL	PI 181434	*Xwgp118‐Xwgp115*	ASR	/	Li et al. (2011)
*Yr46*	*T. aestivum*	4DL	RL6007	*Xgwm165/Xgwm192*	HTAPR	Hexose transporter	Herrera‐Foessel et al. (2011) Moore et al. (2015)
*Yr47*	*T. aestivum*	5BS	AUS28183	*Xgwm234‐Xcfb309*	ASR	/	Bansal et al. (2011)
*Yr48*	*T. aestivum*	5AL	PI 610750	*Xwmc727‐Xcfa2149*	HTAPR	/	Lowe et al. (2011)
*Yr49*	*T. aestivum*	3DS	Chuanmai 18	*Xwgp7321‐Xgwm161*	APR	/	McIntosh (2024)
*Yr50*	*Thinopyrum intermedium*	4BL	CH223	*Xbarc1096‐Xwmc47*	ASR	/	Liu et al. (2013)
*Yr51*	*T. aestivum*	4AL	AUS27858	*Xowm45F3R3‐XSun104*	ASR	/	Randhawa et al. (2014)
*Yr52*	*T. aestivum*	7BL	PI 183527	*Xwgp5258‐Xbarc182*	HTAPR		Ren et al. (2012)
*Yr53*	*T. durum*	2BL	PI 480148	*Xwmc441‐XLRRrev/NLRRrev*_*350*_	ASR	/	Xu et al. (2013)
*Yr54*	*T. aestivum*	2DL	Quaiu 3	*wPt‐667162‐wPt‐667054*	APR	/	Basnet et al. (2013)
*Yr55*	*T. aestivum*	2DL	Frelon, AUS38882	*Xmag4089‐Xmag3385*	ASR	/	McIntosh (2024)
*Yr56*	*T. durum*	2AS	Wollaroi	*Xsun167‐Xsun168*	APR	/	Bansal et al. (2013)
*Yr57*	*T. aestivum*	3BS	AUS91463	*BS00062676‐Xgwm389*	ASR	/	Randhawa et al. (2015)
*Yr58*	*T. aestivum*	3BS	W195	*X3023704‐Xsun476/Xsun533*	APR	/	Chhetri et al. (2016)
*Yr59*	*T. aestivum*	7BL	PI 178759	*Xwgp5175‐Xbarc32*	HTAPR	/	Zhou, Wang, et al. (2014)
*Yr60*	*T. aestivum*	4AL	Almop	*Xwmc313/Xwmc219‐Xwmc776*	ASR	/	Herrera‐Foessel et al. (2015)
*Yr61*	*T. aestivum*	7AS	Pindong 34	*Xwgp5765b‐Xwp5467*	ASR	/	Zhou, Han, et al. (2014)
*Yr62*	*T. aestivum*	4BL	PI 192252	*Xgwm251‐Xgwm192*	HTAPR	/	Lu et al. (2014)
*Yr63*	*T. aestivum*	7BS	AUS27955	*XsunCS_Yr63‐XsunCS_67*	ASR	/	Mackenzie et al. (2023)
*Yr64*	*T. durum*	1BS	PI 331260	*Xgwm413‐Xgdm33*	ASR	/	Cheng et al. (2014)
*Yr65*	*T. durum*	1BS	PI 480016	*Xgwm18‐Xgwm11*	ASR	/	Cheng et al. (2014)
*Yr66*	*T. aestivum*	3DS	VL Gehun 892	*KASP_18087‐KASP_48179*	ASR	/	Bariana et al. (2022)
*Yr67*	*T. aestivum*	7BL	VL Gehun 892	*KASP_37096‐KASP_23397*	ASR	/	Bariana et al. (2022)
*Yr68*	/	4BL	AGG91587WHEA1	*IWB74301‐IWB28394*	APR	/	McIntosh (2024)
*Yr69*	*Thinopyrum ponticum*	2AS	CH7086	*X2AS33‐Xmag3807*	ASR	/	Hou et al. (2016)
*Yr70*	*Aegilops umbellulata*	5DS	IL 393‐4	*Xgwm190‐Xwmc805*	ASR	/	Bansal et al. (2016)
*Yr71*	*T. aestivum*	3DL	Sunco	*Lr24/Sr24; Xgwm114b* *KASP_16434* et al.	APR	/	Bariana et al. (2016)
*Yr72*	*T. aestivum*	2BL	AUS27506/AUS27894	*Xsun481‐IWB12294*	ASR	/	Chhetri et al. (2023)
*Yr73*	*T. aestivum*	3DL	Avocet R	*DArT‐Seg markers*	ASR	/	Dracatos et al. (2016)
*Yr74*	*T. aestivum*	5BL	Avocet R	*DArT‐Seq markers*	ASR	/	Dracatos et al. (2016)
*Yr75*	*T. aestivum*	7AL	Axe	*sunKASP_427‐sunKASP430*	APR	/	Kanwal et al. (2021)
*Yr76*	*T. aestivum*	3AS	Tyee	*Xwmc11‐Xwmc532*	ASR	/	Xiang et al. (2016)
*Yr77*	*T. aestivum*	6DS	PI 322118	*Xbarc54‐Xcfd188*	APR	/	McIntosh (2024)
*Yr78*	*T. aestivum*	6BS	PI 519805/Madsen	*IWA7257‐Xwmc737*	HTAPR	/	Dong et al. (2017)
*Yr79*	*T. aestivum*	7BL	PI 182103	*Xbarc72‐Xwmc335*	HTAPR	/	Feng et al. (2018)
*Yr80*	*T. aestivum*	3BL	Aus27284	*KASP 65624‐KASP 53113*	APR	/	Nsabiyera et al. (2018)
*Yr81*	*T. aestivum*	6AS	Aus27430	*Xgwm459‐KASP_3077*	ASR	/	Gessese et al. (2019)
*Yr82*	*T. aestivum*	3BL	Aus27969	*KASP_73060/KASP_13376‐KASP_8775*	ASR	/	Pakeerathan et al. (2019)
*Yr83*	*S. cereale*	6RL	T‐701/Sub6R(6D)	*Bin FL 0.73‐1.00* (including 33 specific markers)	ASR	/	Li et al. (2020)
*Yr84*	*T. dicoccoides*	1BS	PI 487260	*usw316‐usw317*	ASR	/	Klymiuk et al. (2022)
*Yr85*	*T. aestivum*	1BS	Tres	*IWA2583‐IWA7480*	ASR	/	Feng et al. (2023)
*Yr86*	*T. aestivum*	2AL	Zhongmai 895	*K_AX‐111479506, K_AX‐94907351, K_7127, K_7128, K_7132*	APR	/	Zhu, Cao, et al. (2023)
*Yr87*	*A. sharonensis/Aegilops* *longissima*	6BS	Galil	/	ASR	NLR	Sharma et al. (2024)

Abbreviations: APR, adult‐plant resistance; ASR, all‐stage resistance; HTAPR, high‐temperature adult‐plant resistance.

**FIGURE 1:**
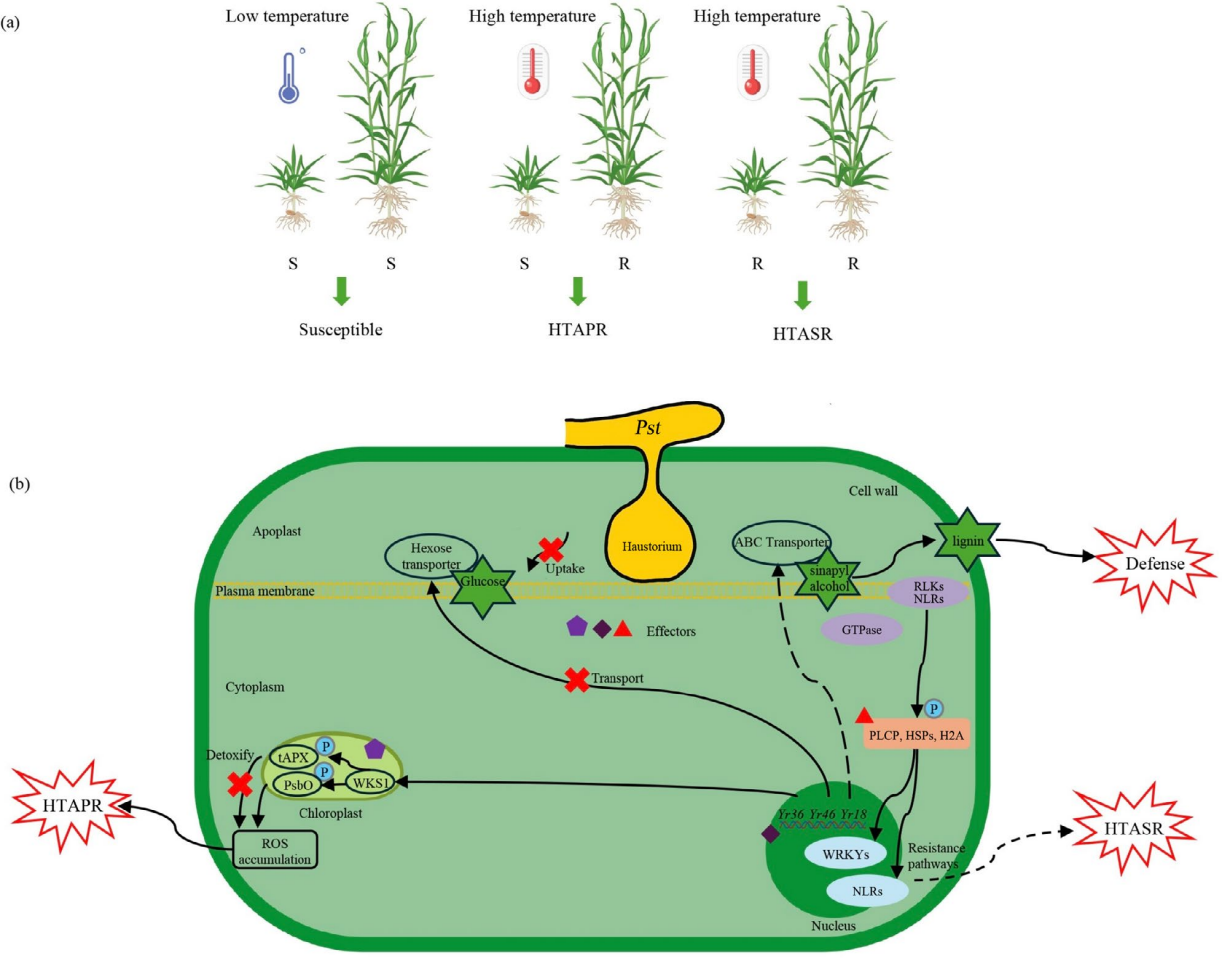
Schematic diagrams of high‐temperature resistance and associated molecular mechanisms in wheat against *Puccinia striiformis* f. sp. *tritici* (Pst). (a) Temperature‐dependent phenotypic responses. Wheat plants exhibit susceptibility to Pst under low‐temperature conditions. In contrast, under high‐temperature conditions, wheat plants develop high‐temperature adult‐plant resistance (HTAPR) and high‐temperature all‐stage resistance (HTASR). (b) A working model of the molecular mechanisms of known high‐temperature resistance in wheat. Solid lines represent well‐studied results, while dotted lines represent the hypothesis proposed. ABC, ATP‐binding cassette; H2A, histone 2A; HSPs, heat shock proteins; NLR, nucleotide‐binding site‐leucine‐rich repeat; P, phosphorylate; PLCP, papain‐like cysteine protease; PsbO, protein subunit of the Photosystem II; RLKs, receptor‐like protein kinases; ROS, reactive oxygen species; tAPX, thylakoid‐associated ascorbate peroxidase; WKS1, wheat kinase‐start 1.

Plant growth and pathogen development are closely related to temperature. In 1968, researchers found that some wheat cultivars shift from being stripe rust disease‐susceptible at 2°C–18°C to resistant at 15°C–24°C (Lewellen and Sharp 1968). As research progressed, wheat temperature‐sensitive resistance to Pst was discovered, which can be divided into high‐temperature adult‐plant (HTAP) resistance and high‐temperature all‐stage (HTAS) resistance according to the growth stage (Figure 1a) (Chen 2005, 2013; Hu et al. 2023; Qayoum and Line 1985; Tao et al. 2018). HTAP resistance in wheat was first described in 1985 (Qayoum and Line 1985). Wheat cultivars with HTAP resistance are susceptible to Pst at the seedling stage and low temperature at the adult‐plant stage but become resistant or more resistant to Pst at high temperatures at the adult‐plant stage (Chen 2007, 2013). An alternative perspective posits that most cases of APR are inherently HTAP resistance, a phenomenon largely attributed to the fact that temperature‐related factors were not adequately incorporated into the identification and characterisation of APR during its initial discovery (Chen and Kang 2017). In addition to HTAP resistance in wheat against Pst, HTAP resistance also exists in wheat against the leaf rust pathogen *Puccinia triticina* f. sp. *tritici* (Ptt) and in barley against the stripe rust pathogen *P. striiformis* f. sp. *hordei* (Upadhaya, Wang, Fatima, et al. 2025; Yan and Chen 2008; Yan et al. 2021). In contrast to HTAP resistance, which is stage‐specific, HTAS resistance manifests throughout the entire growth stage at high temperatures (Gerechter‐Amitai et al. 1984; Hu et al. 2023; Lewellen et al. 1967; Wang et al. 1995). It has also been confirmed that HTAS resistance is regulated by a major gene and multiple minor genes, which in turn reflect its broad‐spectrum and non‐race‐specific characteristics (Lewellen and Sharp 1968; Sharp and Fuchs 1982). Since the discovery of HTAS resistance, most studies have focused on the seedling stage; therefore, it is also called high‐temperature seedling‐plant (HTSP) resistance (Hu et al. 2021; Wang, Tao, An, et al. 2017; Wang, Tao, Tian, et al. 2017). HTSP resistance also exists in wheat against the stem rust pathogen *Puccinia graminis* f. sp. *tritici* and the leaf rust pathogen Ptt (Roelfs 1979; Wang et al. 2025; Zhang et al. 2017).

### 2.2 Conditions of Expression of High‐Temperature Resistance to Pst

The induction of HTAP resistance relies on the synergistic effects of specific plant growth stages and environmental temperature conditions, as elaborated in multiple studies (Chen 2013; Line 2002). HTAP resistance is primarily expressed when plants enter the adult stage, specifically from the tiller stage to the soft dough stage, whereas it is generally not expressed at the seedling stage when plants are mostly susceptible to Pst. Temperature is a critical environmental factor to HTAP resistance. Typical temperature treatments adopted in studies include relatively high diurnal cycles, such as a gradual temperature cycle of 10°C–35°C, or constant temperature cycles of 28°C during the day and 15°C at night, which simulate the high‐temperature characteristics in natural environments during the late growth stage of wheat (Carter et al. 2009; Qayoum and Line 1985). Under these conditions, wheat plants carrying HTAP resistance typically exhibit low infection types and significantly reduced spore production of Pst, in contrast to susceptible plants. However, when temperatures are lower, such as when adult plants are exposed to a low‐temperature cycle of 4°C–20°C, HTAP resistance can be reduced, and wheat plants show susceptibility or reduced resistance (Chen 2013; Chen and Line 1995).

Temperatures that induce HTAS resistance vary among wheat genotypes with diverse genetic backgrounds. In previous studies, the conditions for inducing HTAS resistance were divided into two types. (I) Resistance induced by temperature changes, also known as normal‐high‐normal temperature (NHN) treatment. Wheat plants show susceptibility to Pst when cultivated at normal temperature (N) of 12°C–15°C. In the later stage of latent growth of Pst, the wheat plants are transferred to a relatively high temperature of 18°C–24°C for 12–24 h, and then returned to normal temperature, after which HTAS resistance can be induced (Tao et al. 2018). (II) Resistance induced by consistently high temperatures, also called high‐temperature (H) treatment. Wheat plants show HTAS resistance to Pst when cultivated at a consistent temperature of 10°C–35°C or 15°C–28°C in a daily cycle, which is the same as HTAP resistance. The conditions for this type of HTAS resistance mimic those in natural environments, with the lowest and highest temperatures occurring at 02:00 and 14:00, respectively (Hu et al. 2023). HTAS resistance could be induced throughout the growth stage, and there was no difference in the intensity of resistance at different growth stages (Hu et al. 2012).

### 2.3 Molecular Mapping of the High‐Temperature Resistance Genes

To date, 12 *Yr* genes and numerous quantitative trait loci (QTLs) have been reported to confer HTAP resistance in wheat (Tables 1 and 2). Based on the genetics, HTAP resistance is conferred by one or more major or minor genes (Chen 2013; Liu, Wang, et al. 2020; Mu et al. 2020). A previous study systematically summarised 920 genes/QTLs associated with resistance to leaf rust, stripe rust and stem rust from 170 studies over the past two decades, and found that the number of genes/QTLs in subgenome B was significantly higher than that in subgenomes A and D (Tong et al. 2024). QTL mapping for high‐temperature resistance and transcriptome sequencing revealed that the genes and QTLs associated with HTAP and HTAS resistance to Pst were clustered in the B subgenome of wheat (Table 2). Collectively, these findings highlight the pivotal role of the B subgenome in harbouring genetic loci associated with wheat stripe rust resistance, particularly under high‐temperature conditions, thereby providing valuable information for molecular breeding in wheat.

**TABLE 2:** Characterised genes or quantitative trait loci (QTLs) conferring high‐temperature adult‐plant resistance to *Puccinia striiformis* f. sp. *tritici* in wheat.

Genes/QTL^a^	Mapping population	Flanking (closest) markers	References
*YrCK (2DS)*	Sunco (R) *×* Tasman (S) DH	*Xgdm005‐Xwmc190*	Bariana et al. (2001)
*Yrns‐B1 (3BS)*	Lgst. 79–74 (R) *×* Winzi (S) F_2_	*Xgwm493‐Xgwm1329*	Khlestkina et al. (2006)
*Yrxy1 (7AS)*, *Yrxy2 (2AL)*	Xiaoyan 54 (R) *×* Mingxian 169 (S) F_3_	*Xbarc49‐Xwmc422* *Xgwm794‐Xbarc5*	Zhou et al. (2011)
*YrM1225 (2AS)*	M1225 (R) × AvS (S) F_2‐5_	*IWB44846‐Yr17‐KASP*	Li, Liu, et al. (2023)
*YrM488 (2D)*	M488 (R) × AvS (S) F_2‐5_	*IWA8149‐IWA2792*	Li et al. (2024)
*QYrst.wgp‐6BS.1* *QYrst.wgp‐6BS.2*	Stephens (R) *×* Michigan Amber (S) RIL	*Xbarc101‐Xgdm136* *Xgwm132‐Xgdm113*	Santra et al. (2008)
*QYrlu.cau‐2BS1* *QYrlu.cau‐2BS2* *QYraq.cau‐2BL*	Luke (R) × Aquileja (R) F_3_	*Xwmc154‐Xgwm148* *Xgwm148‐Xabrc167* *Xwmc175‐Xwmc332*	Guo et al. (2008)
*QYrex.wgp‐1BL* *QYrex.wgp‐3BL* *QYrex.wgp‐6AS*	Express (R) × AvS (S)	*Xwmc631‐Xwgp78* *Xgwm299‐Xwgp66* *Xgwm334‐Xwgp56*	Lin and Chen (2009)
*QYrlo.wpg‐2BS*	Louise (R) *×* Penawawa (S) RIL	*Xwmc474–Xgwm148*	Carter et al. (2009)
*QYr‐tem‐5B.1* *QYr‐tem‐5B.2*	Flinor (R) × Mingxian 169 (S) F_3_	*Xwms67‐Xbarc89* *Xwmc235‐Xwms604*	Feng et al. (2010)
*QYrid.ui‐1A* *QYrid.ui‐2B.1* *QYrid.ui‐2B.2* *QYrrb.ui‐3B.1* *QYrid.ui‐3B.2* *QYrid.ui‐4A* *QYrrb.ui‐4B* *QYrid.ui‐5B*	IDO444 (R) *×* Rio Blanco (S) RIL	*X377889–XLMW1* *wPt‐9668–Xgwm429/wPt‐8492* *Xgwm429/X379114–Xbarc91* *X345897–wPt‐3921* *X379646–Xgwm299* *wPt‐2983–wPt‐8275/wPt‐2319* *Xgwm165–Xgwm495* *X63541–Xbarc59*	Chen et al. (2011)
*QYr.uga‐6AS*	Pioneer 26R61 (R) × AGS2000 (S) RIL	*wPt‐671,561–wPt‐7840*	Hao et al. (2011)
*QYrAlt.syau‐3BS*	Alturas (R) × Taichung 29 (S) F_3_	*Xgwm389‐Xbarc238*	Zhao et al. (2012)
*QYrPI192252.wgp‐5BS*	PI 192252 (R) × AvS (S) RIL	*Xgwm335‐IWA6910*	Lu et al. (2014)
*QYrdr.wgp‐1BL.1* *QYrdr.wgp‐1BL.2* *QYrdr.wgp‐1DS* *QYrdr.wgp‐2BL* *QYrdr.wgp‐3AL* *QYrdr.wgp‐5AL* *QYrdr.wgp‐5BL.2* *QYrdr.wgp‐6BL.2*	Druchamp (R) × Michigan Amber (S) RIL	*Xgwm131* *IWA8581* *IWA2268* *IWA7583* *IWA6834* *IWA2558* *IWA6383* *IWA6420*	Hou et al. (2015)
*QYrMa.wgp‐2AS* *QYrMa.wgp‐3BS*	Madsen (R) × AvS (S) RIL	*IWB59733, VENTRIUP‐LN2*, et al. *IWB9910*	Liu et al. (2018)
*QYrel.wgp‐2BS* *QYrel.wgp‐4AL* *QYrel.wgp‐5BS*	Eltan (R) × AvS (S) RIL	*IWB32259* *IWB45728* *IWB56395*	Liu, Wang, et al. (2019)
*QYrsk.wgp‐1BL* *QYrsk.wgp‐3BS* *QYrsk.wgp‐4BL* *QYrsk.wgp‐5AL* *QYrsk.wgp‐6B* *QYrsk.wgp‐7DL*	Skiles (R) × AvS (S) DH	*IWB14436* *IWB41710, IWB67768*, et al. *IWB433, Xbarc163* *IWB47197, IWB53812*, et al. *IWB39258, IWB18*, et al. *IWB7447*	Liu, Yuan, et al. (2019)
*QYrPI197734.wgp‐1AL* *QYrPI197734.wgp‐3BL* *QYrPI197734.wgp‐2A*	PI 197734 (R) × AvS (S) DH	*IWA2015‐IWB6759* *IWB10591‐IWA6297* *IWA5068‐IWA5216*	Liu, Yuan, et al. (2020)
*QYrPI181410.wgp‐1BL* *QYrPI181410.wgp‐4BL* *QYrPI181410.wgp‐5AS* *QYrPI181410.wgp‐5BL.2*	PI 181410× AvS (S) RIL	*IWB6507, IWB13472* *IWB70599, IWB6961*, et al. *IWB47184* *IWB38584, IWB21151*, et al.	Liu, Qie, et al. (2020)
*QYrWW.wgp‐4B* *QYrWW.wgp‐7A.1*	Natural population	*IWB59186* *IWB44935‐IWB11533*	Yao et al. (2024)
*QYrWS.wgp‐1BL* *QyrWS.wgp‐2AL* *QyrWS.wgp‐3AS* *QyrWS.wgp‐3BL*	William Som (R) × AvS (S) RIL	*IWA3998‐IWB7694* *IWA5640‐IWB34603* *IWB63656‐IWB36767* *IWB11270‐IWB22528*	Upadhaya, Wang, Xiang, et al. (2025)

Abbreviations: DH, doubled haploid; R, resistant; RIL, recombinant inbred line; S, susceptible.

^a^ For temporarily named genes, the chromosome information is shown within parentheses. For QTLs, the chromosome information is indicated at the end of its name.

The long arm of chromosome 7B (7BL) is an important region for HTAP resistance, with multiple genes or QTLs characterised under specific temperature regimes and phenotypic assessment stages such as *Yr39*, *Yr52*, *Yr59* and *Yr79*. These genes explain 12.6%–64.2% of the phenotypic variation, which was evaluated using the relative area under disease progress curve (rAUDPC) and infection type (IT), and have been validated across multiple environments (Feng et al. 2018; Lin and Chen 2007; Ren et al. 2012; Zhou, Wang, et al. 2014). Additionally, *Yr2*, *Yr6* and *YrZH84*, which confer the ASR to Pst, were mapped to chromosome 7B (Li, Zheng, et al. 2006; Lupton and Macer 1962; Macer 1966). The dense distribution of these resistance genes and QTLs on 7BL indicates the presence of resistance gene clusters in this region, providing a genetic basis for durable resistance. Chromosome 3BS is another hotspot for HTAP resistance QTLs. For example, the major‐effect QTL *QYrAlt.syau‐3BS* is mapped to 3BS with stable expression across different environments, explaining 34.28%–50.20% of the phenotypic variation, and is distinct from an APR gene *Yr30* and another HTAP resistance gene *Yrns‐B1* on 3BS (Khlestkina et al. 2006; Zhao et al. 2012). *QYrsk.wgp‐3BS* in winter wheat cultivar Skiles is also located on 3BS, with a physical interval of 2.4–18.2 Mb. This QTL explains up to 28.2% of the phenotypic variation, and is tightly linked to the KASP markers *IWB1836* and *IWA7230* (Liu, Yuan, et al. 2019). For most HTAP resistance genes or QTLs, the identification process is as follows: (1) Inoculation at low and high temperatures during the seedling and adult plant stages in the greenhouse using the four‐way test procedure to confirm that a certain germplasm has HTAP resistance (Chen 2013); (2) crossing the HTAP‐resistant cultivar with a susceptible cultivar to construct mapping populations, including F_2_, recombinant inbred line (RIL), and doubled haploid (DH); (3) genotyping the population lines using molecular markers, conducting QTL mapping by integrating phenotypic data from multiple years and sites in fields and in greenhouse; (4) QTLs that were stably expressed, derived from the HTAP‐resistant parent, and not detected at the seedling stage are considered as HTAP resistance QTLs. However, the expression patterns, temperature thresholds and regulatory mechanisms of the HTAP resistance QTLs remain unclear and require further clarification.

As for the breeding application of HTAP resistance genes and QTLs, *Yr48*, *Yr59* and *Yr62* have been introgressed into Chinese elite cultivars via marker‐assisted selection. The resulting lines showed enhanced resistance to Pst and improved agronomic traits (Yang et al. 2021; Zhang et al. 2022; Zhou et al. 2023). Beyond single‐gene applications, QTL combinations also drive durable resistance. The long‐term resistance of winter wheat cultivar Madsen relies on five QTLs with additive effects, and breeding lines carrying all these QTLs exhibit resistance levels comparable to Madsen itself (Liu et al. 2018). Skiles, another winter wheat cultivar, owes its high‐level HTAP resistance to six QTLs, and marker‐assisted selection of lines with multiple QTLs ensures strong resistance across different environments (Liu, Yuan, et al. 2019). In summary, HTAP resistance genes and QTLs, when integrated into elite wheat cultivars through marker‐assisted selection combined with conventional breeding, effectively boost stripe rust resistance. This provides a reliable strategy for breeding wheat cultivars with durable resistance and high adaptability to diverse ecological conditions.

### 2.4 Molecular Mechanism of the High‐Temperature Resistance

In recent years, with the publication of the wheat reference genome and the development of biological technologies, research on the molecular mechanisms of high‐temperature resistance to Pst has rapidly increased. Three HTAP resistance genes, *Yr18*, *Yr36* and *Yr46*, have been cloned (Figure 1b). *Yr18* encodes an ATP‐binding cassette (ABC) transport protein that promotes cell necrosis and inhibits the growth and development of Pst (Krattinger et al. 2009). The ABC transporter directly transports sinapyl alcohol, a key precursor of lignin monomers, across the plasma membrane to the cell wall, where accumulated sinapyl alcohol promotes cell wall lignification and significantly enhances the physical barrier against Pst invasion (Zhang et al. 2024). The *Yr18*‐mediated resistance is durable and non‐race‐specific, and the gene also provides resistance to other diseases or morphological traits, such as leaf rust, stem rust, powdery mildew and leaf tip necrosis, namely *Yr18*/*Lr34*/*Sr57*/*Pm38*/*Ltn1* (Lagudah et al. 2009). The cloned *Yr36* gene is derived from wild emmer wheat ( *Triticum turgidum* subsp. *dicoccoides*) and encodes a WKS1 (*wheat kinase START 1*) protein containing a kinase domain and a START (*steroidogenic acute regulatory protein‐related lipid‐transfer*) domain that exhibits resistance to stripe rust at 25°C–30°C and loses this resistance at 15°C (Fu et al. 2009). Further research on *Yr36* showed that WKS1.1 protein kinase targets and phosphorylates the chloroplast protein tAPX (*thylakoid‐associated ascorbate peroxidase*) and reduces its ability to detoxify peroxide, promoting cell necrosis and enhancing resistance to stripe rust (Gou et al. 2015). WKS1.1 interacts with and phosphorylates an extrinsic member of photosystem II PsbO (*protein subunit of the Photosystem II*) and confers stripe rust resistance by reducing photosynthesis and regulating leaf chlorosis (Wang, Li, et al. 2019). The link between HTAP resistance and growth/development implies a potential functional balance crosstalk. In addition, a recent study revealed that WKS1.1 phosphorylates and activates KAT‐2B (*keto‐acyl thiolase‐2B*), thereby enhancing resistance to Pst through the jasmonate (JA) signalling pathway (Yan et al. 2023). *Yr46* is another cloned HTAP resistance gene, and is a pleiotropic locus that is synonymous with or closely linked to the leaf rust resistance gene, stem rust resistance gene, powdery mildew resistance gene and leaf tip necrosis‐controlling gene *Lr67/Sr55/Pm46/Ltn3* (Forrest et al. 2014; Herrera‐Foessel et al. 2011, 2014; Yao et al. 2024). *Yr46/Lr67* encodes a hexose transporter. The resistant allele differs from the susceptible allele by two amino acid polymorphisms, with the Arg144Gly variation being primarily responsible for the loss of glucose transport activity. Notably, the resistant proteins can form heterodimers with functional susceptible proteins and their homeologs in subgenomes A and B. This binding inhibits glucose uptake and suppresses the growth of Pst and other pathogenic fungi by altering the leaf hexose ratio (Moore et al. 2015). *Yr39* was identified from the spring wheat cultivar Alpowa and is mapped to chromosome 7BL (Lin and Chen 2007). Transcriptome analysis revealed that resistance is activated at approximately 48 hours post‐inoculation (hpi), restricting fungal biomass. Ninety‐nine HTAP‐specific transcripts have been identified, more than half of which are involved in defence and signal transduction, including pathogenesis‐related (PR) proteins, phenylpropanoid biosynthesis genes, protein kinases and *R* gene homologues (Coram et al. 2008). In addition, PR proteins are involved in the HTAP resistance through salicylic acid (SA) and JA signalling pathways (Farrakh et al. 2018). These diverse *R* gene homologues and defence‐related transcripts collectively contribute to race non‐specificity, making it difficult for the pathogen to overcome, which forms the molecular basis for the durability of HTAP resistance (Chen et al. 2013).

As for the HTAS resistance, 21 wheat entries were identified to have HTAS resistance by evaluating 400 Shaanxi landraces and 92 breeding cultivars/breeding lines in 1995 (Wang et al. 1995). The cultivar Xiaoyan 6 (XY6) has maintained its stripe rust resistance and elite agronomic traits since its development in 1975 (Shang 1998). HTAS resistance of XY6 was induced by NHN temperature treatment and persisted throughout the growth stage. Transcriptomic analysis revealed 1395 up‐regulated differentially expressed genes (DEGs) of XY6 in the NHN treatment compared to the N treatment. KEGG enrichment analysis showed that the DEGs were primarily involved in ribosome biogenesis, glycerolipid metabolism pathways and plant–pathogen interactions, while GO enrichment results showed that the DEGs were involved in membrane proteins, ribonucleoside binding proteins and protein kinase activity (Tao et al. 2018). Based on transcriptomic data, a series of transcription factors in the WRKY family were identified and analysed. Through gene silencing mediated by the barley stripe mosaic virus (BSMV), it was found that the transcription factors encoded by *TaWRKY70*, *TaWRKY62* and *TaWRKY45* positively regulated HTAS resistance in XY6 (Wang, Tao, An, et al. 2017; Wang, Tao, Tian, et al. 2017). Nucleotide‐binding site‐leucine‐rich repeat proteins (NLRs) and GTPase proteins have also been demonstrated to be involved in HTAS resistance in XY6 (Hu et al. 2021; Shi et al. 2025; Wang, Tian, et al. 2019). Receptor‐like protein kinases (RLKs) are currently the largest superfamily of molecular recognition receptors identified in plants and play important roles in reproduction, growth and development, immunity and signal transduction (Kanyuka and Rudd 2019; Liang and Zhou 2018). A typical RLK consists of an extracellular ligand domain, transmembrane domain and intracellular kinase domain. Based on differences in the extracellular domains, RLKs can be classified into leucine‐rich repeat (LRR) RLKs, cysteine‐rich RLKs (CRKs), wall‐associated RLKs, plant lectin RLKs, PR5‐like RLKs, epidermal growth factors and receptor‐like cytoplasmic kinases (RLCKs). TaXa21 (*resistant to Xanthomonas oryzae* pv. *oryzae 21*), an LRR‐RLK, perceives the infection signal from Pst through its LRR domain, transmits the signal to the transcription factors TaWRKY76 and TaWRKY62, and initiates HTAS resistance via the ethylene (ETH) pathway (Wang, Wang, et al. 2019). TaCRK10 (*cysteine‐rich receptor‐like kinase 10*), which belongs to the CRK subfamily, interacts with TaH2A.1 (*histone variant*) and activates HTAS resistance by regulating nuclear processes (Wang et al. 2021). TaRIPK (*Pseudomonas syringae pv. maculicola 1‐induced protein kinase*), an RLCK, interacts with and phosphorylates TaPLCP1 (*papain‐like cysteine protease 1*) to activate HTAS resistance (Hu et al. 2022). In summary, existing studies have systematically revealed that the HTAS resistance mechanism involves WRKYs, NLRs, GTPase proteins and members of the RLK family in resistance signal transduction and regulation, which lays a foundation for in‐depth research and utilisation of wheat HTAS resistance (Figure 1b). However, the above‐mentioned molecular mechanisms are limited to XY6; conservation in wheat with different genetic backgrounds and different types of HTAS resistance remains unclear (Hu et al. 2023). Another LRR‐RLK, TaSERK1, perceives the dual signal from Pst and high temperatures, and transmits the signal to TaDJA7 (*DnaJ‐like Protein A7*), a member of the TaHSP40 (*heat shock protein 40*) subfamily, thereby activating HTAS resistance (Shi et al. 2023). Notably, TaHSP90 can form a complex with Lr13 (*leaf rust resistance gene*) and TaRAR1 (*required for Mla12 resistance*) to prevent Lr13 degradation by the 26S proteasome at high temperatures, thereby activating temperature‐sensitive disease resistance responses (Wang et al. 2025). *Lr13* and *Yr27* are allelic variants of the same gene locus (Athiyannan et al. 2022; McDonald et al. 2004). It is hypothesised that heat shock protein (HSP) family genes may play a ‘molecular switch’ role in the HTAS resistance to rust pathogens. Specifically, HSPs may perceive high‐temperature signals and regulate the stability of key disease resistance proteins, thereby participating in the signal pathway of HTAS resistance. This hypothesis not only provides a new research direction for subsequent analysis of the conserved mechanisms of HTAS resistance in wheat with different genetic backgrounds, but also lays a foundation for integrating multiple types of disease resistance‐related genes to construct a more comprehensive regulatory network for high‐temperature rust resistance.

### 2.5 Pst Effectors in Response to High‐Temperature Resistance

Plants have evolved two parallel pathways to defend themselves against biotic stresses (Dangl et al. 2013). One is pathogen‐associated molecular pattern (PAMP)‐triggered immunity (PTI) (Ausubel 2005), and the other is effector‐triggered immunity (ETI) (Jones and Dangl 2006). Plants employ RLKs to sense PAMPs and activate downstream defence responses (Couto and Zipfel 2016). Thereafter, pathogen‐release effectors perform multiple functions, such as inhibiting and interfering with host resistance, evading host recognition and misleading plant defence, thereby promoting the colonisation and growth of pathogens to suppress PTI (Uhse and Djamei 2018). In the arms race between wheat and stripe rust pathogen, Pst secretes effectors that overcome HTAS resistance in wheat. The effector PSTG_01766 targets TaPLCP1 to influence its subcellular localisation and reduce its phosphorylation, further inhibiting the HTAS resistance triggered by the TaRIPK‐TaPLCP1‐TaRPM1 (RLCK‐central hub‐NLR) module (Hu et al. 2022). During the antagonistic interactions and co‐evolution of Pst and wheat HTAS resistance, synergistic interactions exist between the effector proteins of Pst to overcome host resistance. The effector protein PSTG_11208 is recognised by apoplastic TaTLP1 (*thaumatin‐like protein 1*), which induces HTAS resistance in wheat through TaPR1 (*pathogenesis‐related protein 1*). Another effector, PstCEP1 (*Pst candidate effector protein 1*), interacts with PSTG_11208 to prevent the recognition by wheat. PstCEP1 targets TaFd1 (*ferredoxin 1*) and inhibits HTAS resistance in wheat by inhibiting the production of reactive oxygen species (ROS) and interfering with the stability of the photosynthetic system (Bao et al. 2023; Tao et al. 2020). In addition, it is well known that suppressor of the SGT1 (*G2 allele of S‐phase kinase‐associated protein 1*)‐RAR1‐HSP90 module is required for multiple disease responses to viruses, bacteria, oomycetes and fungi in plants, including wheat against Pst (Takahashi et al. 2003; Wang et al. 2015). HSPs are induced by temperature, drought, salinity and biotic stresses, highlighting their multifunctional role in growth, development and stress response networks (Bao et al. 2014; Samakovli et al. 2021; Wang, Ju, et al. 2024). Previous studies have also demonstrated that TaHSPs play a vital role in HTAS resistance to Pst (Tao et al. 2018). The pathogen effector PstSIE1 (*Pst SGT1‐interacting effector 1*) competes with TaRAR1 to bind TaSGT1, disrupts subcomplex formation, further inhibits the resistance mediated by TaHSP90 (Wang, Liu, et al. 2022). Collectively, these findings indicate that Pst employs diverse strategies to counteract HTAS resistance in wheat, thereby providing critical insights into the molecular mechanisms underlying the dynamic interplay between Pst and wheat under high‐temperature conditions.

## 3 Future Directions

Although significant progress has been made in the research on the high‐temperature resistance of wheat to stripe rust, numerous critical challenges remain in mechanism elucidation, gene resource exploration and practical applications. Future studies should focus on the following key areas.

### 3.1 Identifying and Cloning of the High‐Temperature Resistance Genes

To date, only three HTAP resistance genes (*Yr18*, *Yr36* and *Yr46*) have been cloned; however, the molecular nature of most mapped HTAP resistance genes and QTLs remains unclear. Drawing on a comprehensive synthesis of existing references, future efforts should prioritise the targeted cloning of genes and QTLs that exhibit strong breeding potential and clear genetic basis, rather than broad, untargeted screening. First, major‐effect HTAP resistance genes and QTLs with stable expression across multiple environments are key candidates. For instance, for *Yr52*, *Yr59*, *Yr62*, *Yr78* and *Yr79*, in‐depth fine mapping and map‐based cloning have rarely been carried out following the mapping of these HTAP genes. Among QTLs, *QYrAlt.syau‐3BS* stably explains 34.28%–50.20% of phenotypic variation across diverse environments and is distinct from other known resistance loci *Yr30* and *Yrns‐B1* on the same chromosome, while *QYrsk.wgp‐3BS* has a narrow physical interval (2.4–18.2 Mb) and is tightly linked to KASP markers *IWB1836* and *IWA7230*. If map‐based cloning encounters insurmountable barriers for these QTLs, a direct functional investigation of candidate genes within these intervals represents a viable alternative strategy. Specifically, ethyl methanesulfonate‐induced mutagenesis can be employed to generate a mutant library targeting the QTL interval, and subsequent phenotypic screening and co‐segregation analysis between mutant phenotypes and candidate gene mutations enable the identification of causal genes underlying the resistance trait, such as *Yr87/Lr85* and *Pm12/Pm21* (Li, Liu, et al. 2023; Sharma et al. 2024; Zhu, Liu, et al. 2023). Alternatively, reverse genetics approaches, including comparative transcriptomics and quantitative proteomics, can prioritise functionally relevant genes by integrating multi‐omics datasets (Li, Hua, et al. 2023). Gene functional validation will also help gain a deeper understanding of disease resistance mechanisms. Secondly, resistance gene clusters in chromosomal hotspots represent a critical focus. Comparative genomic analysis of the clusters revealed tandemly duplicated resistance genes, evolutionary relationships and potential functional redundancies that are essential for understanding durable resistance. Research on HTAS remains limited, with most studies concentrating on a single germplasm and a few cloned genes. To address this gap, future studies should first screen wheat germplasm resources for novel HTAS resistance with a particular focus on landraces, especially those from regions with high spring temperatures (Hu et al. 2023). Once promising accessions have been identified, advanced techniques, including genome‐wide association studies (GWAS), QTL fine mapping, bulked segregant analysis (BSA)‐seq and long‐reading sequencing, should be applied to clone HTAS resistance genes.

### 3.2 Deciphering Molecular Regulatory Networks and Signal Pathways of High‐Temperature Resistance

Previous studies have revealed partial regulatory mechanisms of HTAP resistance genes, such as *Yr36*, which regulates ROS accumulation and photosynthesis via phosphorylation of the chloroplast proteins tAPX and PsbO, and *Yr18* mediating cell necrosis through ABC transporters to inhibit pathogen expansion, as well as *Yr46*, which inhibits glucose uptake and suppresses the growth of Pst (Fu et al. 2009; Krattinger et al. 2009; Moore et al. 2015; Wang, Li, et al. 2019). However, the upstream and downstream signalling pathways of most HTAP resistance genes remain unclear. As for HTAS resistance, although WRKYs, NLRs and RLKs in cultivar XY6 have been found to participate in resistance through several signalling pathways. Nevertheless, how HTAS resistance‐specific temperature response patterns initiate signals, and the differences and similarities in the molecular regulatory networks between HTAP and HTAS resistance, remain to be elucidated.

Future studies should systematically dissect the cross‐integration mechanisms of high‐temperature signals and pathogen infection signals using multi‐omics technologies such as transcriptomics, proteomics and metabolomics. On one hand, studies are needed to clarify how high temperatures activate the specific pathways of HTAP and HTAS resistance through temperature‐sensing elements such as HSPs and calcium signalling molecules. On the other hand, studies are also needed to reveal the interaction networks between Pst effectors and core regulatory factors in both resistance types, for example, WKS1.1 in HTAP resistance and TaCRK10 in HTAS resistance, and to further elucidate the molecular basis of the temperature–pathogen–host tripartite interaction (Wang, Li, et al. 2019; Wang et al. 2021). In addition, for identified pathways such as SA, JA and ethylene, further exploration is needed to reveal differences in metabolic accumulation and key enzyme activity regulation related to high‐temperature resistance, thereby improving the regulatory networks and uncovering the molecular basis of synergy or differentiation of HTAP and HTAS resistance (Hu et al. 2022; Wang, Tao, Tian, et al. 2017; Wang et al. 2021).

### 3.3 Unveiling the Genetic Basis of Multigene Synergistic Resistance

Multigene synergistic resistance in wheat to stripe rust is key for sustainable disease control, with its genetic basis centered on the major gene and minor QTL collaborative framework. In HTAP resistance, major and minor genes/QTLs are densely located on chromosomes 7BL and 3BS, and the wheat B subgenome, enriched with more resistance loci, serves as the core for synergistic regulation. These genes/QTLs enhance resistance stability through additive effects. Combining the ASR and HTAP genes to provide resistance throughout the growth period is an effective strategy for improving broad‐spectrum resistance during breeding (Bariana et al. 2022). The multigene synergy of HTAS resistance was reflected in a multipathway defence network. Taking XY6 as an example, TaWRKY transcription factors and RLK family genes activate different defence branches by sensing high‐temperature and pathogen signals to form a synergy; however, the conservation of this mechanism in wheat with different genetic backgrounds needs to be determined.

### 3.4 Studying Effector‐Mediated Resistance Suppression and Counteraction Mechanisms in the Pathogen

Pathogen effectors that interfere with host resistance are critical for stripe rust development (Petre et al. 2014). Previous studies have found that three Pst effectors inhibit HTAS resistance in the wheat cultivar XY6 (Bao et al. 2023; Hu et al. 2022; Tao et al. 2020). Advances in genomics, such as long‐read sequencing and comparative genomic analysis, have facilitated the identification of Pst effectors and clarified their roles in wheat–Pst interactions, which is fundamental to understanding the evolution of Pst virulence (Guan et al. 2025). A series of Pst effectors have been reported to inhibit or interfere with the host immune response (Duan et al. 2024; Pan et al. 2024; Wang, Tang, et al. 2022; Wang, Liu, et al. 2024; Wu et al. 2022). However, investigations of effector genes paired with permanently named *Yr* genes, including HTAP resistance genes, remain scarce. Future studies should be prioritised on identifying key *Pst* effectors that target high‐temperature resistance pathways and clarify their mechanisms of inhibiting host resistance‐related proteins. Concurrently, studies should decipher how hosts evolve countermeasures against effectors via HTAP and HTAS resistance genes, revealing the co‐evolutionary dynamics of effectors and resistance genes. This will advance the plant immunity theory and identify targets for novel disease control strategies.

### 3.5 Breeding for High‐Temperature Resistance Genes

Breeding utilisation of high‐temperature resistance genes is currently limited by inadequate marker precision and unstable resistance expression (Chen 2013). Future efforts should focus on developing gene sequence‐based functional markers to improve the gene selection efficiency. Additionally, gene‐editing technologies could optimise resistance gene expression patterns to address the challenge of co‐improving resistance and agronomic traits (Li, Li, et al. 2021). In light of the research on temperature adaptability in the context of climate change, wheat cultivars capable of stably expressing resistance across diverse temperature gradients should be developed (Zhu et al. 2021). This approach aims to enhance the regional adaptability of resistant wheat cultivars. Meanwhile, the utilisation rate of characterised molecular markers should also be improved. Well‐characterised markers can act as efficient tools for marker‐assisted selection in wheat high‐temperature resistance breeding, not only enabling rapid identification and pyramiding of favourable resistance loci but also reducing the reliance on time‐consuming and labour‐intensive phenotypic evaluation (Mourad et al. 2024).

## 4 Conclusions

Wheat stripe rust, caused by the obligate biotrophic fungus Pst, is a major biotic stressor in global wheat‐producing regions. High‐temperature resistance has emerged as a core breakthrough in the sustainable management of wheat stripe rust, owing to its race‐non‐specificity and durability. This review summarises the research progress on the high‐temperature resistance of wheat against Pst. In terms of the genetic basis, genes and QTLs for HTAP resistance are clustered in chromosomal regions. HTAS resistance has been confirmed to be co‐regulated by major genes and minor QTLs; however, relevant studies remain limited to the germplasm with a narrow genetic background, and its genetic basis and molecular networks remain to be clarified. At the pathogen interaction level, Pst can secrete effectors to target wheat resistance proteins, thereby suppressing high‐temperature resistance, revealing the dynamic interplay in the wheat–Pst–high temperature tripartite interaction.

Nevertheless, gaps remain in current research: the cloning and functional verification of HTAS resistance genes are limited, the targeting mechanism of Pst effectors in HTAP resistance pathways remains to be elucidated, the genetic networks and regulatory logic of multigene synergistic resistance are unclear and the marker accuracy and application efficiency of high‐temperature resistance genes in molecular breeding require further improvement. Future integrated research focusing on ‘gene mining‐network deciphering‐effector interaction‐breeding application’ will not only improve the theoretical system of plant high‐temperature‐mediated disease resistance but also provide key support for breeding wheat varieties with both durable rust resistance and excellent agronomic traits under the context of climate change, ultimately safeguarding global wheat production security.

## Author Contributions

**Xinqian Lyu:** data curation, writing – original draft, software. **Guocheng Li:** data curation, writing – original draft. **Chang Su:** data curation. **Xujun Luo:** data curation. **Yuankang Zhu:** data curation. **Tao Liu:** data curation, writing – review and editing. **Yuxiang Li:** data curation, writing – review and editing. **Xiaoping Hu:** conceptualization, writing – review and editing. **Chengyun Li:** conceptualization, writing – review and editing. **Yangshan Hu:** conceptualization, data curation, funding acquisition, writing – review and editing, writing – original draft, software.

## Funding

This work was supported by Yunnan Agricultural Joint Project (202301BD070001‐179) and Opening Foundation of State Key Laboratory for Biology of Plant Disease and Insect Pests (SKLOF202413).

## Conflicts of Interest

The authors declare no conflicts of interest.

## Data Availability

The authors have nothing to report.
